# 5-Chloro-2-(3,4,5-trimeth­oxy­phen­yl)-1,3-benzothia­zole

**DOI:** 10.1107/S1600536812039372

**Published:** 2012-09-29

**Authors:** Sammer Yousuf, Shazia Shah, Nida Ambreen, Khalid M. Khan, Shakil Ahmad

**Affiliations:** a HEJ Research Institute of Chemistry, International Center for Chemical and Biological Sciences, University of Karachi, Karachi 75270, Pakistan

## Abstract

In the title compound, C_16_H_14_ClNO_3_S, the dihedral angle between the almost-planar benzothia­zole ring system [maximum deviation = 0.012 (3) Å] and the aromatic ring of the trimeth­oxy­phenyl group is 15.56 (6)°. In the crystal, the mol­ecules are arranged into layers parallel to the *bc* plane, held together only by weak van der Waals forces.

## Related literature

For the biological activites of benzothia­zole compounds, see: Chohan *et al.* (2003 ▶); Hutchinson *et al.* (2002 ▶); Chua *et al.* (1999 ▶); Burger & Sawhney (1968 ▶); Palmer *et al.* (1971 ▶). For the crystal structures of related benzothia­zole derivatives, see: Yousuf *et al.* (2012*a* ▶,*b* ▶).
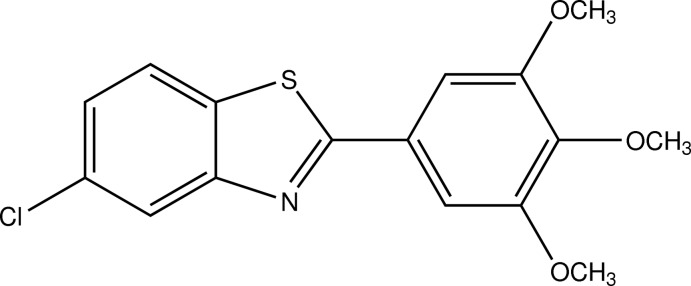


## Experimental

### 

#### Crystal data

C_16_H_14_ClNO_3_S
*M* *_r_* = 335.79
Triclinic, 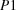

*a* = 4.0656 (6) Å
*b* = 7.7855 (11) Å
*c* = 12.2420 (17) Å
α = 96.263 (3)°
β = 91.380 (3)°
γ = 97.228 (3)°
*V* = 381.84 (9) Å^3^
*Z* = 1
Mo *K*α radiation
μ = 0.40 mm^−1^
*T* = 273 K
0.52 × 0.15 × 0.09 mm

#### Data collection

Bruker SMART APEX CCD diffractometer
Absorption correction: multi-scan (*SADABS*; Bruker, 2000 ▶) *T*_min_ = 0.820, *T*_max_ = 0.965
4277 measured reflections
2816 independent reflections
2621 reflections with *I* > 2σ(*I*)
*R*_int_ = 0.014

#### Refinement

*R*[*F* ^2^ > 2σ(*F* ^2^)] = 0.034
*wR*(*F* ^2^) = 0.080
*S* = 1.07
2816 reflections
202 parameters
3 restraints
H-atom parameters constrained
Δρ_max_ = 0.14 e Å^−3^
Δρ_min_ = −0.16 e Å^−3^
Absolute structure: Flack (1983 ▶), with 1402 Friedel pairs
Flack parameter: 0.12 (6)

### 

Data collection: *SMART* (Bruker, 2000 ▶); cell refinement: *SAINT* (Bruker, 2000 ▶); data reduction: *SAINT*; program(s) used to solve structure: *SHELXS97* (Sheldrick, 2008 ▶); program(s) used to refine structure: *SHELXL97* (Sheldrick, 2008 ▶); molecular graphics: *SHELXTL* (Sheldrick, 2008 ▶); software used to prepare material for publication: *SHELXTL*, *PARST* (Nardelli, 1995 ▶) and *PLATON* (Spek, 2009 ▶).

## Supplementary Material

Crystal structure: contains datablock(s) global, I. DOI: 10.1107/S1600536812039372/rz5005sup1.cif

Structure factors: contains datablock(s) I. DOI: 10.1107/S1600536812039372/rz5005Isup2.hkl

Supplementary material file. DOI: 10.1107/S1600536812039372/rz5005Isup3.cml

Additional supplementary materials:  crystallographic information; 3D view; checkCIF report

## Figures and Tables

**Fig. 1.**
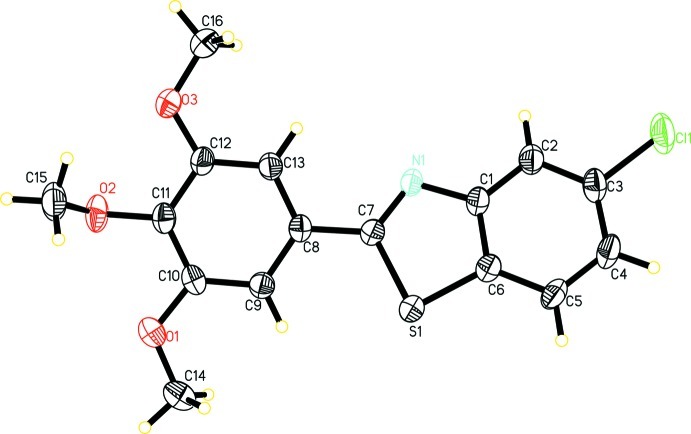
The molecular structure of title compound with displacement ellipsoids drawn at 30% probability level.

**Fig. 2.**
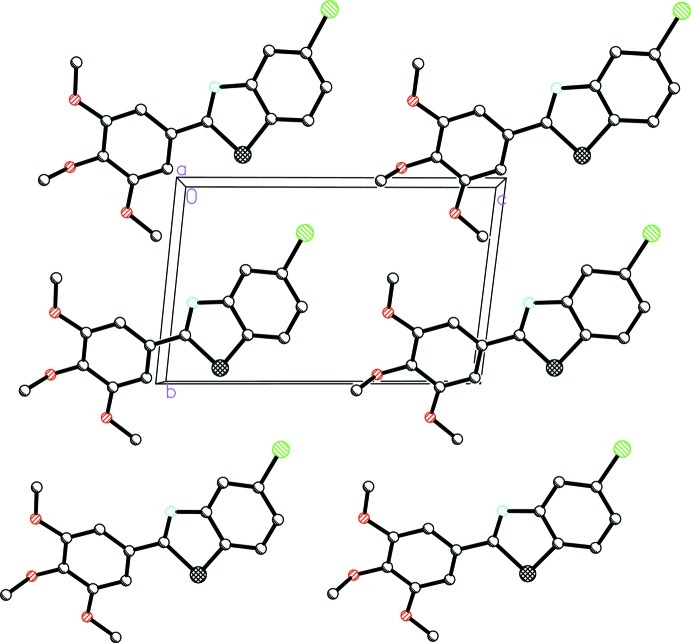
The crystal packing of the title compound. Hydrogen atoms are omitted for clarity.

## supplementary crystallographic information

### Comment

Benzothiazole is a well known class of sulfur- and nitrogen-containing heterocyclic aromatic molecules with a broad range of biological activities, such as antimicrobial, antitumoral, antimalarial and antitubercular (Chohan *et al.*, 2003; Hutchinson *et al.*, 2002; Chua *et al.*, 1999; Burger & Sawhney, 1968; Palmer *et al.*, 1971). The title compound is a benzothiazole derivative synthesized as a part of our ongoing project on bioactive hetereocyclic compounds.

The molecular structure of the title compound (Fig. 1) is similar to that reported for the recently published compounds 5-chloro-2-phenyl-1,3-benzothiazole (Yousuf *et al.*, 2012*a*) and 2-(5-chloro-1,3-benzothiazol-2-yl)-4-methoxyphenol (Yousuf *et al.*, 2012*b*) with the difference that the phenyl or p-methoxyphenol group is replaced by a trimethoxyphenyl group. The dihedral angle between the almost planar benzothiazole ring system (S1/N1/C1–C7) and the benzene ring of the trimethoxyphenyl group (C8–C13) is 15.56 (6)°. Bond lengths and angles are unexceptional. In the crystal structure the molecules are arranged into layers parallel to the *bc* plane (Fig. 2) held together only by weak van der Waals forces.

### Experimental

A mixture of 2-amino-4-cholorobenzenethiol (0.159 g, 1 mmol), 3,4,5-trimethoxybenz-aldehyde (0.196 g, 1 mmol), sodium metabisulfite (0.2 g) and *N*,*N*-dimethylformamide (10 ml) was refluxed for 2 h in a round-bottomed flask. The completion of reaction was monitored by TLC. After cooling the mixture to room temperature, cold water was added to obtain a white precipitate. Crystallization from ethanol afforded crystals of the title compound (0.298 g, 88.9% yield) found suitable for X-ray diffraction studies.

### Refinement

H atoms were positioned geometrically with C—H = 0.96 or 0.93 Å, and constrained to ride on their parent atoms with *U*_iso_(H) = 1.2*U*_eq_(C) or 1.5*U*_eq_(C) for methyl H atoms. A rotating group model was applied to methyl groups.

### Crystal data

**Table:**

C_16_H_14_ClNO_3_S	*Z* = 1
*M**_r_* = 335.79	*F*(000) = 174
Triclinic, *P*1	*D*_x_ = 1.460 Mg m^−^^3^
Hall symbol: P 1	Mo *K*α radiation, λ = 0.71073 Å
*a* = 4.0656 (6) Å	Cell parameters from 2110 reflections
*b* = 7.7855 (11) Å	θ = 1.7–25.5°
*c* = 12.2420 (17) Å	µ = 0.40 mm^−^^1^
α = 96.263 (3)°	*T* = 273 K
β = 91.380 (3)°	Plate, colourless
γ = 97.228 (3)°	0.52 × 0.15 × 0.09 mm
*V* = 381.84 (9) Å^3^	

### Data collection

**Table:**

Bruker SMART APEX CCD diffractometer	2816 independent reflections
Radiation source: fine-focus sealed tube	2621 reflections with *I* > 2σ(*I*)
Graphite monochromator	*R*_int_ = 0.014
ω scans	θ_max_ = 25.5°, θ_min_ = 1.7°
Absorption correction: multi-scan (*SADABS*; Bruker, 2000)	*h* = −4→4
*T*_min_ = 0.820, *T*_max_ = 0.965	*k* = −9→9
4277 measured reflections	*l* = −14→14

### Refinement

**Table:**

Refinement on *F*^2^	Secondary atom site location: difference Fourier map
Least-squares matrix: full	Hydrogen site location: inferred from neighbouring sites
*R*[*F*^2^ > 2σ(*F*^2^)] = 0.034	H-atom parameters constrained
*wR*(*F*^2^) = 0.080	*w* = 1/[σ^2^(*F*_o_^2^) + (0.0384*P*)^2^ + 0.0343*P*] where *P* = (*F*_o_^2^ + 2*F*_c_^2^)/3
*S* = 1.07	(Δ/σ)_max_ < 0.001
2816 reflections	Δρ_max_ = 0.14 e Å^−^^3^
202 parameters	Δρ_min_ = −0.16 e Å^−^^3^
3 restraints	Absolute structure: Flack (1983), with 1402 Friedel pairs
Primary atom site location: structure-invariant direct methods	Flack parameter: 0.12 (6)

### Special details

**Table:**

Geometry. All e.s.d.'s (except the e.s.d. in the dihedral angle between two l.s. planes) are estimated using the full covariance matrix. The cell e.s.d.'s are taken into account individually in the estimation of e.s.d.'s in distances, angles and torsion angles; correlations between e.s.d.'s in cell parameters are only used when they are defined by crystal symmetry. An approximate (isotropic) treatment of cell e.s.d.'s is used for estimating e.s.d.'s involving l.s. planes.
Refinement. Refinement of *F*^2^ against ALL reflections. The weighted *R*-factor *wR* and goodness of fit *S* are based on *F*^2^, conventional *R*-factors *R* are based on *F*, with *F* set to zero for negative *F*^2^. The threshold expression of *F*^2^ > σ(*F*^2^) is used only for calculating *R*-factors(gt) *etc*. and is not relevant to the choice of reflections for refinement. *R*-factors based on *F*^2^ are statistically about twice as large as those based on *F*, and *R*-factors based on ALL data will be even larger.

### Fractional atomic coordinates and isotropic or equivalent isotropic displacement parameters (Å^2^)

**Table:**

	*x*	*y*	*z*	*U*_iso_*/*U*_eq_	
S1	0.46803 (14)	0.91830 (7)	0.20220 (5)	0.05768 (18)	
Cl1	0.9092 (2)	0.29518 (10)	0.41340 (7)	0.0924 (3)	
O1	−0.0441 (5)	1.1670 (2)	−0.14201 (16)	0.0662 (5)	
O2	0.0285 (5)	0.9542 (3)	−0.32262 (15)	0.0703 (6)	
O3	0.2731 (5)	0.6534 (2)	−0.31396 (14)	0.0670 (5)	
N1	0.4815 (5)	0.6095 (3)	0.10078 (16)	0.0510 (5)	
C1	0.5921 (6)	0.6053 (3)	0.2087 (2)	0.0483 (6)	
C2	0.6876 (8)	0.4599 (4)	0.2507 (2)	0.0603 (7)	
H2A	0.6804	0.3539	0.2070	0.072*	
C3	0.7927 (7)	0.4782 (4)	0.3588 (2)	0.0616 (7)	
C4	0.8097 (7)	0.6314 (4)	0.4269 (2)	0.0651 (8)	
H4A	0.8842	0.6378	0.4999	0.078*	
C5	0.7142 (8)	0.7758 (4)	0.3852 (2)	0.0651 (8)	
H5A	0.7238	0.8813	0.4296	0.078*	
C6	0.6036 (6)	0.7615 (3)	0.2761 (2)	0.0499 (6)	
C7	0.4065 (5)	0.7623 (3)	0.08620 (18)	0.0443 (5)	
C8	0.2955 (5)	0.8118 (3)	−0.01945 (19)	0.0436 (5)	
C9	0.1638 (6)	0.9681 (3)	−0.0251 (2)	0.0479 (5)	
H9A	0.1328	1.0395	0.0388	0.057*	
C10	0.0797 (6)	1.0154 (3)	−0.1272 (2)	0.0495 (6)	
C11	0.1223 (6)	0.9078 (3)	−0.2224 (2)	0.0523 (6)	
C12	0.2466 (6)	0.7500 (3)	−0.21641 (19)	0.0498 (6)	
C13	0.3346 (6)	0.7029 (3)	−0.11455 (19)	0.0496 (5)	
H13A	0.4198	0.5984	−0.1100	0.060*	
C14	−0.1064 (8)	1.2779 (4)	−0.0479 (3)	0.0675 (7)	
H14A	−0.2101	1.3735	−0.0699	0.101*	
H14B	0.0992	1.3218	−0.0085	0.101*	
H14C	−0.2508	1.2140	−0.0014	0.101*	
C15	0.2896 (9)	1.0281 (4)	−0.3824 (2)	0.0788 (9)	
H15A	0.2008	1.0805	−0.4421	0.118*	
H15B	0.4177	0.9390	−0.4109	0.118*	
H15C	0.4291	1.1154	−0.3349	0.118*	
C16	0.4033 (8)	0.4926 (4)	−0.3130 (2)	0.0722 (8)	
H16A	0.4046	0.4368	−0.3869	0.108*	
H16B	0.2677	0.4185	−0.2695	0.108*	
H16C	0.6258	0.5138	−0.2820	0.108*	

### Atomic displacement parameters (Å^2^)

**Table:**

	*U*^11^	*U*^22^	*U*^33^	*U*^12^	*U*^13^	*U*^23^
S1	0.0794 (4)	0.0528 (4)	0.0413 (3)	0.0137 (3)	−0.0038 (3)	0.0030 (3)
Cl1	0.1234 (7)	0.0817 (5)	0.0763 (6)	0.0143 (5)	−0.0310 (5)	0.0349 (4)
O1	0.0760 (13)	0.0611 (11)	0.0673 (12)	0.0246 (10)	−0.0039 (9)	0.0170 (9)
O2	0.0682 (13)	0.0986 (15)	0.0487 (11)	0.0164 (11)	−0.0162 (9)	0.0271 (10)
O3	0.0937 (14)	0.0661 (12)	0.0416 (10)	0.0188 (10)	−0.0110 (9)	0.0008 (8)
N1	0.0684 (13)	0.0473 (11)	0.0368 (10)	0.0066 (9)	−0.0086 (9)	0.0065 (8)
C1	0.0522 (14)	0.0512 (14)	0.0404 (14)	−0.0003 (11)	−0.0047 (10)	0.0100 (11)
C2	0.0778 (18)	0.0532 (15)	0.0498 (15)	0.0072 (13)	−0.0096 (13)	0.0102 (12)
C3	0.0693 (18)	0.0652 (18)	0.0525 (17)	0.0033 (14)	−0.0078 (13)	0.0252 (14)
C4	0.0791 (19)	0.079 (2)	0.0365 (14)	0.0035 (16)	−0.0100 (13)	0.0120 (13)
C5	0.091 (2)	0.0690 (18)	0.0334 (13)	0.0088 (16)	−0.0050 (13)	−0.0003 (12)
C6	0.0539 (14)	0.0541 (15)	0.0407 (13)	0.0045 (11)	−0.0001 (11)	0.0041 (11)
C7	0.0453 (13)	0.0460 (13)	0.0406 (13)	0.0009 (10)	0.0003 (10)	0.0056 (10)
C8	0.0436 (13)	0.0464 (13)	0.0405 (12)	0.0005 (10)	−0.0017 (9)	0.0106 (10)
C9	0.0500 (13)	0.0459 (13)	0.0467 (13)	0.0018 (10)	−0.0002 (10)	0.0058 (10)
C10	0.0435 (13)	0.0495 (13)	0.0579 (15)	0.0050 (10)	−0.0029 (11)	0.0189 (11)
C11	0.0505 (13)	0.0602 (15)	0.0467 (14)	0.0046 (11)	−0.0084 (10)	0.0139 (11)
C12	0.0533 (14)	0.0553 (14)	0.0403 (13)	0.0045 (11)	−0.0052 (10)	0.0076 (10)
C13	0.0573 (14)	0.0473 (13)	0.0446 (13)	0.0069 (11)	−0.0048 (10)	0.0084 (10)
C14	0.0626 (16)	0.0536 (15)	0.088 (2)	0.0140 (12)	0.0002 (14)	0.0110 (13)
C15	0.099 (2)	0.087 (2)	0.0558 (17)	0.0127 (18)	−0.0040 (16)	0.0311 (15)
C16	0.093 (2)	0.0726 (19)	0.0516 (16)	0.0205 (16)	−0.0017 (15)	−0.0021 (14)

### Geometric parameters (Å, º)

**Table:**

S1—C6	1.731 (3)	C5—H5A	0.9300
S1—C7	1.756 (2)	C7—C8	1.466 (3)
Cl1—C3	1.750 (3)	C8—C13	1.388 (3)
O1—C10	1.368 (3)	C8—C9	1.397 (3)
O1—C14	1.410 (4)	C9—C10	1.388 (3)
O2—C11	1.375 (3)	C9—H9A	0.9300
O2—C15	1.403 (3)	C10—C11	1.387 (4)
O3—C12	1.354 (3)	C11—C12	1.395 (3)
O3—C16	1.420 (3)	C12—C13	1.389 (3)
N1—C7	1.294 (3)	C13—H13A	0.9300
N1—C1	1.391 (3)	C14—H14A	0.9600
C1—C2	1.387 (4)	C14—H14B	0.9600
C1—C6	1.388 (3)	C14—H14C	0.9600
C2—C3	1.368 (4)	C15—H15A	0.9600
C2—H2A	0.9300	C15—H15B	0.9600
C3—C4	1.372 (4)	C15—H15C	0.9600
C4—C5	1.378 (4)	C16—H16A	0.9600
C4—H4A	0.9300	C16—H16B	0.9600
C5—C6	1.387 (4)	C16—H16C	0.9600
			
C6—S1—C7	88.87 (11)	C8—C9—H9A	120.4
C10—O1—C14	118.2 (2)	O1—C10—C11	115.7 (2)
C11—O2—C15	114.8 (2)	O1—C10—C9	124.0 (2)
C12—O3—C16	118.1 (2)	C11—C10—C9	120.4 (2)
C7—N1—C1	110.9 (2)	O2—C11—C10	119.6 (2)
C2—C1—C6	120.0 (2)	O2—C11—C12	120.0 (2)
C2—C1—N1	124.9 (2)	C10—C11—C12	120.3 (2)
C6—C1—N1	115.1 (2)	O3—C12—C13	124.8 (2)
C3—C2—C1	117.5 (3)	O3—C12—C11	115.6 (2)
C3—C2—H2A	121.2	C13—C12—C11	119.5 (2)
C1—C2—H2A	121.2	C8—C13—C12	120.0 (2)
C2—C3—C4	123.7 (3)	C8—C13—H13A	120.0
C2—C3—Cl1	118.0 (2)	C12—C13—H13A	120.0
C4—C3—Cl1	118.3 (2)	O1—C14—H14A	109.5
C3—C4—C5	118.8 (3)	O1—C14—H14B	109.5
C3—C4—H4A	120.6	H14A—C14—H14B	109.5
C5—C4—H4A	120.6	O1—C14—H14C	109.5
C4—C5—C6	119.1 (3)	H14A—C14—H14C	109.5
C4—C5—H5A	120.5	H14B—C14—H14C	109.5
C6—C5—H5A	120.5	O2—C15—H15A	109.5
C5—C6—C1	120.9 (2)	O2—C15—H15B	109.5
C5—C6—S1	129.3 (2)	H15A—C15—H15B	109.5
C1—C6—S1	109.74 (19)	O2—C15—H15C	109.5
N1—C7—C8	124.4 (2)	H15A—C15—H15C	109.5
N1—C7—S1	115.40 (17)	H15B—C15—H15C	109.5
C8—C7—S1	120.09 (17)	O3—C16—H16A	109.5
C13—C8—C9	120.5 (2)	O3—C16—H16B	109.5
C13—C8—C7	118.58 (19)	H16A—C16—H16B	109.5
C9—C8—C7	120.9 (2)	O3—C16—H16C	109.5
C10—C9—C8	119.2 (2)	H16A—C16—H16C	109.5
C10—C9—H9A	120.4	H16B—C16—H16C	109.5
			
C7—N1—C1—C2	179.2 (2)	S1—C7—C8—C9	−15.0 (3)
C7—N1—C1—C6	−0.9 (3)	C13—C8—C9—C10	−1.5 (3)
C6—C1—C2—C3	−0.4 (4)	C7—C8—C9—C10	176.2 (2)
N1—C1—C2—C3	179.5 (3)	C14—O1—C10—C11	177.3 (2)
C1—C2—C3—C4	−0.3 (4)	C14—O1—C10—C9	−3.4 (3)
C1—C2—C3—Cl1	179.2 (2)	C8—C9—C10—O1	−178.5 (2)
C2—C3—C4—C5	0.4 (5)	C8—C9—C10—C11	0.7 (3)
Cl1—C3—C4—C5	−179.1 (2)	C15—O2—C11—C10	100.9 (3)
C3—C4—C5—C6	0.1 (5)	C15—O2—C11—C12	−81.8 (3)
C4—C5—C6—C1	−0.8 (4)	O1—C10—C11—O2	−2.6 (3)
C4—C5—C6—S1	179.7 (2)	C9—C10—C11—O2	178.1 (2)
C2—C1—C6—C5	0.9 (4)	O1—C10—C11—C12	−179.9 (2)
N1—C1—C6—C5	−179.0 (2)	C9—C10—C11—C12	0.7 (3)
C2—C1—C6—S1	−179.5 (2)	C16—O3—C12—C13	−0.7 (4)
N1—C1—C6—S1	0.6 (3)	C16—O3—C12—C11	179.0 (2)
C7—S1—C6—C5	179.4 (3)	O2—C11—C12—O3	1.6 (3)
C7—S1—C6—C1	−0.09 (18)	C10—C11—C12—O3	178.9 (2)
C1—N1—C7—C8	177.3 (2)	O2—C11—C12—C13	−178.8 (2)
C1—N1—C7—S1	0.8 (3)	C10—C11—C12—C13	−1.5 (3)
C6—S1—C7—N1	−0.43 (19)	C9—C8—C13—C12	0.8 (3)
C6—S1—C7—C8	−177.11 (19)	C7—C8—C13—C12	−177.0 (2)
N1—C7—C8—C13	−13.6 (3)	O3—C12—C13—C8	−179.7 (2)
S1—C7—C8—C13	162.74 (17)	C11—C12—C13—C8	0.7 (3)
N1—C7—C8—C9	168.6 (2)		
